# Modality-dependent bottom-up attention and eye gaze direction affect auditory spatial discrimination

**DOI:** 10.3758/s13414-026-03305-9

**Published:** 2026-07-09

**Authors:** René Šebeňa, Jyrki Ahveninen, Virginia Best, Barbara Shinn-Cunningham, Norbert Kopčo

**Affiliations:** 1 Department of Psychology, Faculty of Arts, P. J. Šafárik University, Košice, Slovak Republic; 2 Athinoula A. Martinos Center for Biomedical Imaging, Department of Radiology, Massachusetts General Hospital, Harvard Medical School, Boston, MA USA; 3 Department of Speech, Language and Hearing Sciences, Boston University, Boston, MA USA; 4 Neuroscience Institute, Carnegie Mellon University, Pittsburgh, PA USA; 5 Institute of Computer Science, Faculty of Science, P. J. Šafárik University, Jesenná 5, 040 01 Košice, Slovak Republic

**Keywords:** Bottom-up attention, Auditory spatial discrimination, EEG, Crossmodal perception

## Abstract

**Abstract:**

A combined behavioral and EEG experiment examined the effects of intramodal and crossmodal cueing on auditory spatial discrimination. On each trial, the participant fixated a neutral location and was presented with an auditory target (two broadband “buzzes” with a slight relative spatial offset) from one of two locations (central or lateral). The target was preceded by a cue varying in its validity (presented from the same or a different location than the target with a 50% probability) and modality (auditory or visual, in separate blocks). The participant’s task was to indicate the direction of the target shift, irrespective of the noninformative cue. Behavioral results showed that valid auditory cues improved discrimination sensitivity relative to invalid cues, while visual cue validity had no effect. Moreover, discrimination responses were biased away from the gaze direction for both cue modalities, with an additional bias away from the invalid auditory cues. EEG analysis identified neural correlates of the behavioral effects; specifically, both cue validity and modality modulated target-elicited auditory N1 and N2 components, while the more posteriorly distributed P3 correlated weakly with response biases. Finally, an auditory cue-evoked posterior contralateral positivity (ACOP) was consistent with a visual (eye-centered) rather than an auditory (head-centered) reference frame, suggesting that auditory signals are converted into a visual representation prior to directing bottom-up attention.

**Open practices statement:**

The data supporting the findings of this study are openly available on Zenodo [10.5281/zenodo.17035882]. The repository includes trial-level behavioral data and raw EEG data associated with the present studies. The studies reported in this manuscript were not preregistered.

**Supplementary Information:**

The online version contains supplementary material available at 10.3758/s13414-026-03305-9.

## Introduction

Real-world environments are often rich, challenging our sensory systems as our brain only can process limited amounts of information. Attention helps prioritize objects, events, or areas in complex scenes, shifting the processing resources to the relevant stimuli (Gazzaniga et al., 2002; Johnson & Proctor, 2004; Posner, 1980). To achieve this, attention can be directed voluntarily, by our goals, or involuntarily, captured by sudden events in the environment (Monsell & Driver, 2000).

Some previous behavioral studies showed that an auditory orienting cue that precedes the stimulus can improve reaction times and accuracy for an auditory spatial discrimination task at the cued location (Sach et al., 2000; Spence & Driver, 1997). However, other studies reported either a cuing benefit restricted to a subregion of auditory space (Maier et al., 2010) or no effect of cuing on localization accuracy (Kopčo et al., 2001). On the other hand, preceding stimuli that direct attention away from the target can have detrimental effects, such as when irrelevant auditory stimuli that share characteristics with the target (“flankers”) cause slower or less accurate responses to the target (Chan et al., 2005).

In addition to “intramodal” cueing, there is also evidence that “crossmodal” cueing can affect orienting of auditory spatial attention (Spence & Driver, 1997). One previous study showed that, in an auditory spatial discrimination task, performance improved when the gaze followed a visual cue to the target location, but not when an auditory cue was used and the eyes did not move (Maddox et al., 2014). However, the study could not separate the effect of visually guided attention from the effect of the eye gaze shifts.

Recent EEG studies have begun to investigate the neural mechanisms underlying intramodal and crossmodal involuntary spatial orienting (Störmer et al., 2019). Several studies showed the importance of natural spatial cues in EEG studies of sound localization and auditory attention (Baumgartner et al., 2017; Deng et al., 2019; Ito et al., 2019). In particular, unnatural spatial cues tend to reduce the neural markers of spatial auditory attention, as spatial selective attention networks are only partially engaged by these impoverished cues (Deng et al., 2019). Therefore, instead of only using simple binaural cues to simulate space (Maddox et al., 2014; Sach et al., 2000), the current study was performed in a virtual auditory environment created using full Head-Related Transfer Functions (HRTFs; Carlile, 1996).

Multiple studies showed that prominent lateralized sounds, when used as cues for attentional orienting in a visual or auditory discrimination task, trigger an enlarged contralateral positive potential in the visual cortex (Feng et al., 2014; McDonald et al., 2013). This auditory-evoked component, labeled as contralateral occipital positivity (ACOP), was observed within the interval of 200–450 ms after the cue onset even when the cues were spatially and temporally nonpredictive of subsequent target events. These crossmodal cueing effects likely result from the orienting of visual attention towards the location of the salient sound, which occurs even when the targets are auditory. However, since visual and auditory space are encoded in different reference frames (RFs, eye-centered vs. head-centered, respectively; Groh & Sparks, 1992; Kopčo et al., 2019), this process must rely on an alignment of the auditory and visual signals in the same RF. The current study examined the neural correlates of such auditory cuing with focus on ACOP-like responses and their RFs.

Here, we report the results of a combined behavioral and EEG experiment exploring how bottom-up attention affects auditory spatial discrimination. We examined both beneficial and detrimental effects of stimulus-driven orienting, with the cue provided in either the auditory or visual modality to contrast intramodal and crossmodal mechanisms. We used a similar approach to that of Maddox et al. (2014), employing a cue that varied in location (valid or invalid) and modality (auditory or visual) and an auditory spatial discrimination task, but in our design the participants fixated on a neutral location to eliminate the influence of gaze shifts. The main goal was to determine whether directing covert bottom-up orienting attention affects performance in a spatial discrimination task, and whether the effect depends on the modality of the attentional cue when the gaze direction is fixed at a neutral location. We hypothesized that valid cues would improve performance while invalid cues would have a negative effect. We also expected that the effect would be stronger for the intra-modal auditory cue than the cross-modal visual cue, as the effect of eye gaze was expected to be eliminated by keeping fixation at a neutral location equidistant from valid and invalid cues in the current study. EEG activity was recorded during the experiment and target-evoked and cue-evoked ERPs were analyzed. We expected neural responses to targets to correlate with the behavioral effects of the cue validity and modality. For the auditory cue-elicited responses, we expected the ACOP-related response strength to correlate with cued discrimination performance. Finally, since the spatial setup of the experiment differed in eye-centered and head-centered RFs, we aimed to identify the RF in which the ACOP-related responses are encoded.

## Methods

### Participants

Thirteen subjects (ages 20–38 years, nine men) participated in the experiment. All participants had normal hearing by self-report, and all provided written informed consent as approved by the Ethical Committee of the P. J. Šafárik University in Košice.

### Setup

The experiment was performed in a double-walled sound-proof booth with electromagnetic shielding (Eckel Laboratories, Cambridge, MA). The subject was seated on a chair in front of a 24-inch LCD monitor and a keyboard. The head was positioned at the start of each experimental block by adjusting the chair and screen so the eyes were aligned with the center of the screen at a distance of 42 cm to ensure the correct visual cue and fixation point (FP) azimuths of 0°, ±12.5°, and ±25°. The subject was instructed to keep the head fixed throughout the session. However, the head position was not stabilized by any support or monitored, to prevent any interference with the EEG cap/wiring. Also, the subject was instructed to keep the eyes fixated at the FP during trials. The experiment was controlled by a PC (placed outside the booth) using MATLAB (MathWorks, Natick, MA) with the Psychtoolbox 3 extension (Brainard, 1997). Auditory stimuli were delivered using Etymotic Research (Elk Grove Village, IL) ER-1 insert headphones connected to a Datapixx system (VPixx Technologies, Saint-Bruno, Quebec Canada), with a programmable USB audio device (Texas Instruments TLV320AIC3256EVM-U). Subjects followed on-screen instructions and entered their responses using the PC keyboard. Subjects wore a 32-electrode EEG cap (Biosemi ActiveTwo, Amsterdam, Netherlands) with additional electrodes at the temples to monitor eye-gaze direction. EEG data were recorded during the whole experiment on a separate data acquisition PC.

### Stimuli

**Fig. 1:**
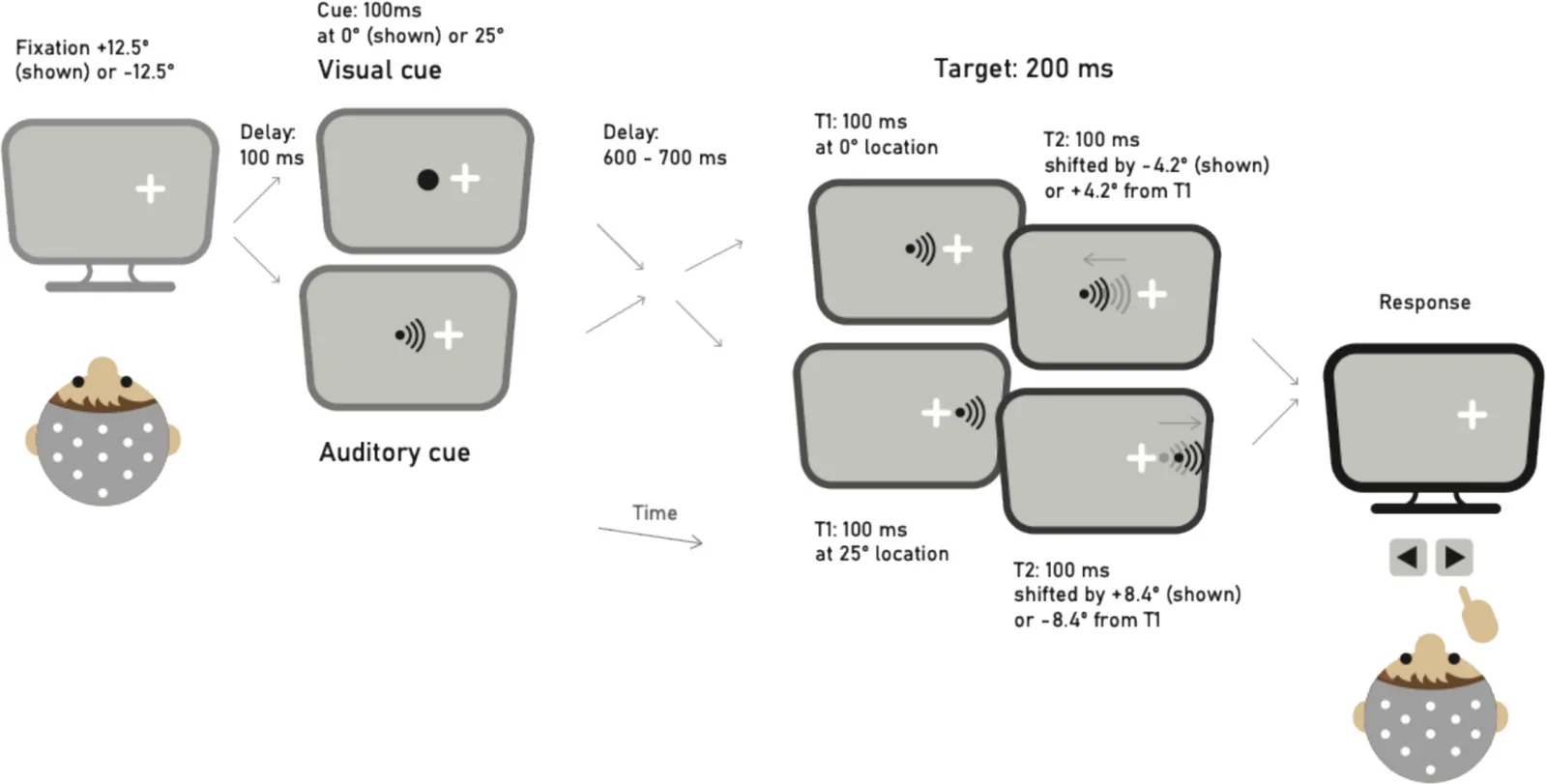
Experimental setup and trial structure. Each trial started with the onset of the FP, followed by the presentation of a cue (visual or auditory) and a target (consisting of two sounds, T1 and T2). The cue validity and target shift directions were randomly chosen on each trial (50% probability), while the FP location and cue modality were fixed within a block. In the figure the FP is located at +12.5° and the cue and target are presented in the right hemifield. In half of the blocks, the setup was left–right mirrored, with the FP at −12.5° and the stimuli presented in the left hemifield

The auditory stimuli were 100-ms broad-band “buzz” sounds with 5-ms on/off ramps, created as pulses (single 1 s among 0 s in the digital signal) presented repetitively at a repetition rate of 170 Hz. They were sampled at 48828 Hz and presented at average level of 70 dB SPL. The target (Fig. 1) consisted of two such buzz sounds (referred to as T1 and T2), presented without any temporal gap from two neighboring locations. T1 locations were 0° or ±25° while T2 was shifted from T1 by ±4.2°, for the 0° T1, or by ±8.4°, for the ±25° T1 (the T2 offsets at each location were determined prior to the experiment as average just-noticeable differences in location for a group of five pilot subjects). The auditory cue consisted of one such buzz, preceding the target from the azimuth of 0° or ±25°. The auditory stimulus locations were simulated using KEMAR HRTFs (Gardner & Martin, 1994) interpolated by CSOUND functions (Lazzarini & Carty, 2008). Visual stimuli included a fixation point (FP), shown during the whole duration of a trial as a white cross at the azimuth of ±12.5° on the black computer screen, and a visual cue, shown as a 100-ms white dot at the azimuths of 0° or ±25°, subtending 0.8°. All stimuli were presented at an elevation of 0°.

### Procedure

The structure of individual trials is illustrated in Fig. 1. Each trial started with an FP appearing on the screen at +12.5° (shown in Fig. 1) or −12.5°. Subjects were required to hold their gaze at the FP throughout the entire trial (monitored off-line based on EOG) and they generally kept the eyes steady even during the inter-trial intervals (confirming this, an analysis of the EOGs did not show any systematic eye movements in response to FP; data not shown). After 100 ms, the auditory or visual cue was presented with equal probability from one of the two locations +12.5° or −12.5° re. the FP location (thus, if the FP was at +12.5°, the cue could come from 0°, as shown in Fig. 1, or from +25°). The target, presented 600–700 ms after the cue offset (chosen randomly on each trial from a uniform distribution), consisted of two components, T1 and T2. Like the cue, the T1 component was presented with equal probability from +12.5° or −12.5° re. the FP (on valid trials, the cue and T1 locations were the same, as illustrated by the upper alternative for T1 in Fig. 1 where both cue and T1 are at 0°; on invalid trials, the cue and T1 came from different locations, as illustrated by the lower alternative for T1 in Fig. 1 where the T1 is at +25°). Finally, T2 was presented immediately after T1 and offset from it with equal probability to the left or to the right by 4.2° (if T1 was at 0°) or by 8.4° (if T1 was at ±25°). The subject’s task was to indicate whether the target moved to the left or right (i.e., whether T2 was to the left or right of T1) using the keys “1” or “2” on the numeric keypad. No feedback was provided. The next trial started 700 ms after the response. Note that since the cue and target locations were offset relative to the FP, the setup was always left–right symmetrical in the eye-centered RF (targets and cues always ±12.5° from the FP), while in the head-centered RF the cues and targets were presented either in the left hemifield (−25° and 0° from the straight ahead orientation) or the right hemifield (+25° and 0°).

The experiment consisted of two sessions, performed on two separate days, with an additional practice session performed immediately before Session 1. The practice session started with a block of 30 no-cue trials with the subject fixating at +12.5° and target presented at [T1, T2] of [0°, ±12°] or [25°, 25°±18°]. This block served to ensure that the subjects could perform the task. Each subject had to achieve performance above 50% correct without feedback, with the block repeated until this criterion was reached. The practice session proceeded with four 20-trial blocks, one for each combination of FP location (+12.5° or −12.5°) and cue modality (visual or auditory). The subject had to reach an average of 60% correct responses across these four blocks. All subjects exceeded this criterion.

The two experimental sessions consisted of 20 blocks each, with 40 trials per block. Within each block, fixation location and cue modality were held constant, while target position, target shift direction, and cue validity varied randomly from trial to trial with equal probability (i.e., the cue was valid on 50% of trials). Subjects were free to take breaks between blocks. The first session began with a block of trials using left fixation and a visual cue, followed by a block with right fixation and a visual cue. Next came a block with left fixation and an auditory cue, followed by a block with right fixation and an auditory cue. This sequence of four blocks was repeated five times throughout the session. In the second session, the order of the blocks was reversed, starting with right fixation and auditory cue. The choice of fixing the eyes and cue modality within a block was made, respectively, to minimize potentially distracting eye movements and uncertainty that the randomized cue modality could cause otherwise. The order in which the subjects were exposed to auditory and visual cues was counterbalanced across sessions, but not randomized within session, to prevent the effects of prolonged exposure to the same cue or to the same FP location which could otherwise occur (e.g., if the same cue modality is randomly selected on two consecutive blocks. In total, 1,600 trials were performed in the experiment, 50 for each combination of 2 [target locations] × 2 [shift directions] × 2 [cue validities] × 2 [cue modalities] × 2 [fixations]. Each session lasted approximately 40 min.

### Behavioral data analysis

For each condition with FP at +12.5°, there was a left–right symmetric condition with FP at −12.5°. Since the results in the corresponding left–right symmetric conditions were largely mirrored, data collected with −12.5° FP were mirror-flipped and combined with the data collected with the +12.5° FP. Moreover, unless stated otherwise, the data are presented as if the FP was always at +12.5°. Sensitivity index *d′* and criterion location c were used to evaluate performance, computed by considering separately the percent correct data for the target shift towards and away from the FP, and assigning the correct response when the target moved away as a ”hit” and an incorrect response when the target moved towards FP as a “false alarm.” A repeated-measures analysis of variance (ANOVA) in SPSS software was used to evaluate statistical significance, with α = 0.05 and all reported *p* values corrected for potential violations of the sphericity assumption using the Box–Geissler–Greenhouse epsilons.

### EEG acquisition and preprocessing

EEG data were recorded at a sampling rate of 4096 Hz from 32 scalp electrodes positioned in the standard 10/20 configuration. Flat-type electrodes with individual leads/connectors were placed on the earlobes for reference. Two electrodes were placed above and below the left eye and two additional ones at the outside corners of each eye to measure the EOG. The processing of the EEG data involved epoching the trials between −0.5 to 1 s relative to the onset of the auditory/visual cue condition and −1 to 1 s relative to the onset of the target stimulus. For ERP analysis, data were filtered using a 1-Hz high-pass and 40-Hz low-pass filter, and the signals were referenced against the two electrodes on the subject's earlobes and down-sampled to 256 Hz. The number of trials per condition was 50. For each electrode, all trials with raw EEG values exceeding 100 μV were rejected to reduce noise. EOG data were epoched between −0.9 s to 0.2 s relative to the onset of target T1. This epoch interval allowed us to evaluate the eye gaze direction during the presentation of FP, cue and target stimulus. EEG trials contaminated with eye blink artifacts (voltage threshold ± 100 μV relative to pretrial baseline), determined by vertical EOG data analysis, were rejected. Trials where the subject did not hold his/her gaze steady at the FP, as determined from the horizontal EOG data analysis, were also rejected. In total, 8.7% of trials were rejected.

### Target ERP analysis

To quantify the amplitudes of individual ERP components, we defined time windows based on the grand-average ERP, obtained by averaging across all conditions, electrodes, and subjects. The grand average ERP showed a standard response consisting of the components of P1 (peak at 39 ms), N1 (102 ms), P2 (188 ms), N2 (254 ms), and P3 (359 ms), referenced to the onset of target T1. The time ranges over which temporal components were considered were determined from the mid-points between the peaks and troughs of the grand average potentials: P1 (16–70 ms), N1 (74–145 ms), P2 (148–219 ms), N2 (223–305 ms), and P3 (309–453 ms). Based on previous studies (Feng et al., 2014; McDonald et al., 2013), the analysis focused on two topographic regions. To analyze the early ERP components (P1, N1, P2), data from four fronto-central channels (Cz, Fc1, Fc2, Fz) were averaged. To examine the later ERP components (N2 and P3), responses were averaged across seven posterior electrodes (Pz, CP1, CP2, P3, P4, PO3, PO4). Averaged ERP components from selected channels were then compared across different conditions using repeated measures ANOVA, with all reported *p* values Box–Greenhouse–Geissler corrected for potential violations of the sphericity assumption.

### Auditory cue ERP analysis

The auditory cue-elicited ERP analysis focused on parieto-occipital orienting effects, similar to the ACOP effects reported in previous studies (Feng et al., 2014; McDonald et al., 2013). These components were analyzed in relation to behavioral discrimination accuracy. The orienting-related component was quantified as the mean amplitude over eight posterior electrode sites (P7/P8, PO3/PO4, P3/P4, O1/O2) within the 300–400 interval after cue onset, similar to the selection in the previous studies (Feng et al., 2014; McDonald et al., 2013). The analysis was performed in head-centered and eye-centered RFs. For the head-centered RF analysis, ERP waveforms were collapsed across auditory stimulus location (left, right) and hemisphere of recording (left, right) to obtain ERPs recorded on the contralateral and ipsilateral hemispheres relative to the FP and peripheral auditory stimulus location. Averaged ERP hemispheric difference (contra − ipsi relative to fixation) components from selected channels were then averaged and compared across different conditions using repeated-measures ANOVAs with the factors of discrimination accuracy (correct/incorrect), validity (valid/invalid), cue position (peripheral/central), and fixation (left/right). For the eye-centered RF analysis, the data were recoded so that the cortical hemispheres were referenced as contralateral versus ipsilateral relative to the cue location encoded in the eye-centered RF (i.e., left vs. right re. fixation).

## Results and discussion

The results are presented in three parts. First, the behavioral data analysis examines how cue validity, cue modality, and fixation direction influenced covert bottom-up orienting in spatial discrimination. Next, an analysis of target-elicited ERPs aims at identifying the ERP components that correlate with the behavioral effects. Finally, the auditory cue-elicited ERP analysis links behavioral performance and the ACOP-like responses and their RF.

### Behavioral performance

#### Results

The effect of cueing on spatial discrimination was analyzed in terms of sensitivity index *d′* and criterion location *c*. Based on the results of Maddox et al. (2014), we hypothesized that covert bottom-up orienting of attention to the target location would improve the discrimination sensitivity on valid trials vs. invalid trials. We also expected that the effect would be stronger for the auditory cue than the visual cue, as eye gaze was fixed to a neutral location in the current study.

**Fig. 2:**
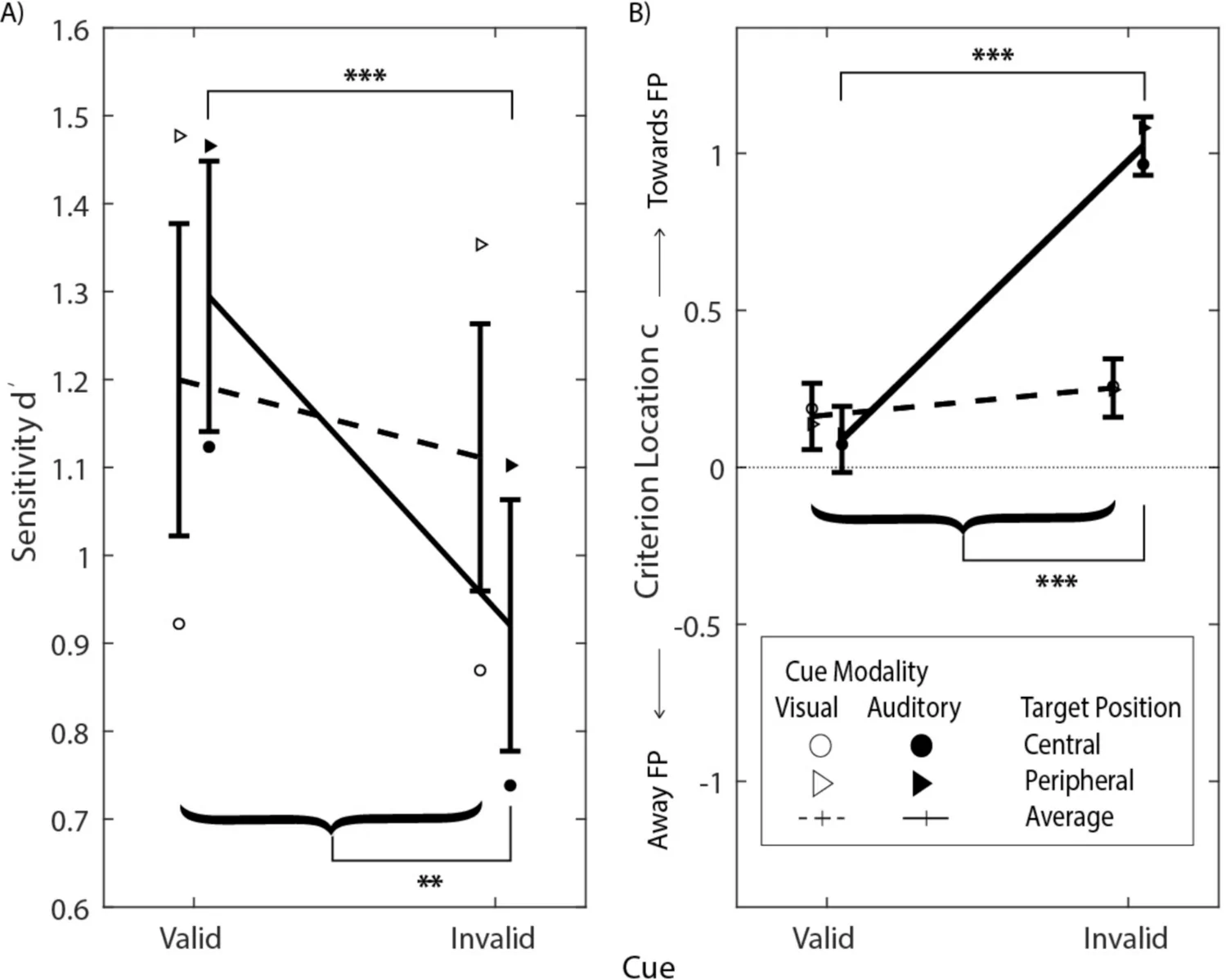
Behavioral performance. Across-subject mean (±SEM) sensitivity *d′* (**A**) and criterion location *c* (**B**) in responses as a function of cue validity, plotted separately for the visual cue (dashed lines) and auditory cue (solid lines). Lines show data averaged across target locations, while symbols show performance separately for the central versus peripheral target location. All data are averaged across left and right fixations. Note that a shift of criterion location “towards FP” corresponds to responses being biased “away from FP” and vice versa. Horizontal lines connect conditions with significant post hoc pairwise differences (significance levels: **p < 0.01, ***p < 0.001), while curly brackets covering pairs of conditions indicate that an average value across the conditions was used in significance testing

Figure 2A shows the *d′* as a function of cue validity, separately for the visual versus auditory cue (dashed vs. solid lines, respectively), collapsed across the central versus peripheral target locations (additionally, the circles and triangles aligned with each corresponding error bar show the central and peripheral target data separately). Valid auditory cues resulted in improved discrimination sensitivity compared to invalid auditory cues (solid line), while the visual cues had a negligible validity effect (dashed line). Confirming this, a repeated-measures ANOVA on the *d′* data with the factors of cue modality (visual, auditory), cue validity (valid, invalid), target location (central, peripheral) and fixation location (left, right) found a significant interaction of cue modality and cue validity, *F*(1,13) = 6.513, *p* = 0.024, with post hoc *t* tests showing a significant difference between the auditory valid versus invalid cues, *t*(13) = 4.28, *p* = 0.001, but not between the visual valid and invalid cues, *t*(13) = 1.54, *p* = 0.146. Since no visual cuing effect was observed, an average of the visual valid and invalid cue values was computed and used as a no-cue reference estimate for determining whether the auditory cuing effect was mainly driven by the valid or the invalid cue. Pairwise comparisons to this reference found that the invalid auditory cue caused a significant decrease in sensitivity, *t*(13) = 3.76, *p* = 0.002, while the improvement caused by the valid auditory cue was not statistically significant, *t*(13) = −1.79, *p* = 0.095, suggesting that the observed auditory cuing effect was driven mainly by a detrimental effect of the invalid cue. The ANOVA also found a weakly significant Target Location × Fixation Location interaction, *F*(1,13) = 2.971, *p* = 0.046, as well as a significant main effect of target location, *F*(1,13) = 13.497, *p* < 0.001. This main effect reflected better performance for the peripheral targets than for central targets (triangles vs. circles in Fig. 2A), while the interaction indicated that the central vs. peripheral difference was slightly stronger with the right fixation (central vs. peripheral *d′* of 0.91 vs. 1.64) than the left fixation (0.99 vs. 1.26). Since the difference between central and peripheral targets is likely due to the larger T1–T2 target separation used at the peripheral location, and the modulation by FP was weak and did not affect the effect of cuing, which is of primary interest here, these effects of target and fixation locations were not further considered. No other main effects or interactions were significant.

The analysis of the criterion placement examined whether there were biases in discrimination responses due to the relative location of the targets re. the gaze direction, and possibly also re. the cue location. While gaze direction-related biases were not analyzed by Maddox et al. (2014), biases away from the FP were previously reported for studies of absolute localization and motion perception (Brimijoin, 2018; Cui et al., 2010). Cue location-related biases were expected mainly for the auditory cue, where the similarity of the cue and target stimuli may have induced the perception of stimulus motion, even though the interval between the cue and the target was relatively long.

Figure 2B plots the criterion location *c* in a format similar to Fig. 2A, with positive bias values corresponding to criterion placement in the direction towards the FP (i.e., representing a subjects’ tendency to perceive the target as moving away from the FP). For the visual cues, there was a slight positive bias, independent of cue validity (dashed line), while for the auditory cue, there was no bias when it was valid and a large positive bias when it was invalid (solid line). These effects were very similar for the central and peripheral target locations (symbols) and independent of fixation location (not shown). Confirming these observations, repeated-measures ANOVAs performed on the criterion data, with the same factors as described above for sensitivity, found a significant Cue Modality × Validity interaction, *F*(1,13) = 23.945, *p* < 0.001, with post hoc analysis showing a strong effect of the validity of auditory cue, *t*(13) = −4.941, *p* < 0.001, and no effect of validity of the visual cue, *t*(13) = −1.229, *p* = 0.241. Finally, *t* tests found that the criterion locations were significantly different from zero for the visual cue, *t*(13) = 2.25, *p* = 0.04, as well as for the auditory invalid cue, *t*(13) = 5.026, *p* < 0.001, but not for the auditory valid cue, *t*(13) = 1.07, *p* = 0.30.

#### Discussion

The *d′* results support the hypothesis that bottom-up orienting affects auditory spatial discrimination sensitivity when it is controlled by the intramodal auditory cue, while no change in sensitivity is induced by the crossmodal visual cue. However, importantly, the auditory cuing effect appears to arise because invalid cues reduce sensitivity, by distracting listeners’ attention, not because valid cues enhance sensitivity. The null visual cue results confirm that the visually induced improvements observed in the study of Maddox et al. (2014) were driven by directing the eye gaze, not attention, to the target location. On the other hand, the lack of an auditory cuing effect in that previous study, compared to the mainly distracting effect of the invalid auditory cue in the current study, is likely to be driven by the experimental differences. Specifically, the effect observed here may have been driven by the realistic HRTF-based spatial simulation and the use of similar sounds for the cue and target, both of which may have increased the potency of the auditory cue.

The criterion results for the visual cue show that listeners had a tendency to perceive the sounds as moving away from the fixation, independent of cue validity. This bias might be related to the bias away from FP reported in static localization (Cui et al., 2010). However, the bias observed here is in relative localization of two consecutive sounds, suggesting that the bias either grows with time (since T2 is perceived as further away from FP than T1) or that it is enhanced by the presentation of T1. Alternatively, previous studies also reported motion perception distortions observed relative to the head, suggesting that frontal sources are perceived as moving faster than lateral sources (Brimijoin, 2018). The current results indicate that such distortions might also depend on the target location re. the gaze fixation.

The criterion results for the auditory cue show that there is a strong preference to hear the target as moving away from the FP when the cue is invalid, while there is no bias for the valid cue. Both of these results can be explained by assuming that there is a flanker-effect-like interference from the cue-to-T1 motion direction (Chan et al., 2005) that affects the ability to determine the direction of motion from T1 to T2. In the visual flanker phenomenon, the perception of the direction of an arrow is affected when the arrow is surrounded by multiple arrows pointing in the opposite direction. In the current study, for the invalid auditory cue, the apparent motion from the cue to T1 covers 25° and thus might dominate over the near-detection-threshold T1–T2 separation of 4–8°, inducing the observed bias. For example, consider a trial in which participants fixate the +12.5° FP and an invalid auditory cue is played from a central location (0°) followed by the target sound presented from the peripheral location (+25° T1 followed by +25°±8° T2). The proposed mechanism predicts that the large rightward cue-to-T1 apparent motion interferes with the difficult small location change from T1 to T2, biasing the subjects to report the cue-to-T1 direction (which in this scenario is the same direction as away from FP). This interference hypothesis is also consistent with the no bias re. FP observed for the valid cue, as in this case the cue and T1 are at the same location, thus not causing any interference with the T1-to-T2 direction perception. On the contrary, having the cue aligned with T1 appears to counteract the away-from-FP bias observed with visual cues. An alternative to the flanker explanation is that the effect might be related to the visual apparent motion inertia illusion (Anstis & Ramachandran, 1987), leading to the perception that the target continues along the cue-to-target trajectory, even if the physical motion does not. Another related observation is that cue predictivity has asymmetric effect on visual reorienting driven by valid vs. invalid cues, possibly due to validity-dependent speed of reorienting to the cue (Doricchi et al., 2010, 2020; Lasaponara et al., 2011, 2017).

Whatever the mechanism, the bias effect of the auditory cue might have been enhanced in the current study by the fact that the cue and target were identical sounds. However, it is likely that it would be also observed if the cue and target differed. Finally, these biases might also be related to the auditory localization aftereffect induced by a preceding adaptor. Even though auditory spatial discrimination studies with a preceding adaptor did not report any similar biases in perception (Getzmann, 2004; Lingner et al., 2018; Maier et al., 2010), localization studies do show clear adaptor-induced biases (Andrejková et al., 2023; Laback, 2023) and the previous discrimination studies did not examine criterion location.

### Target-elicited ERPs

#### Results

**Fig. 3:**
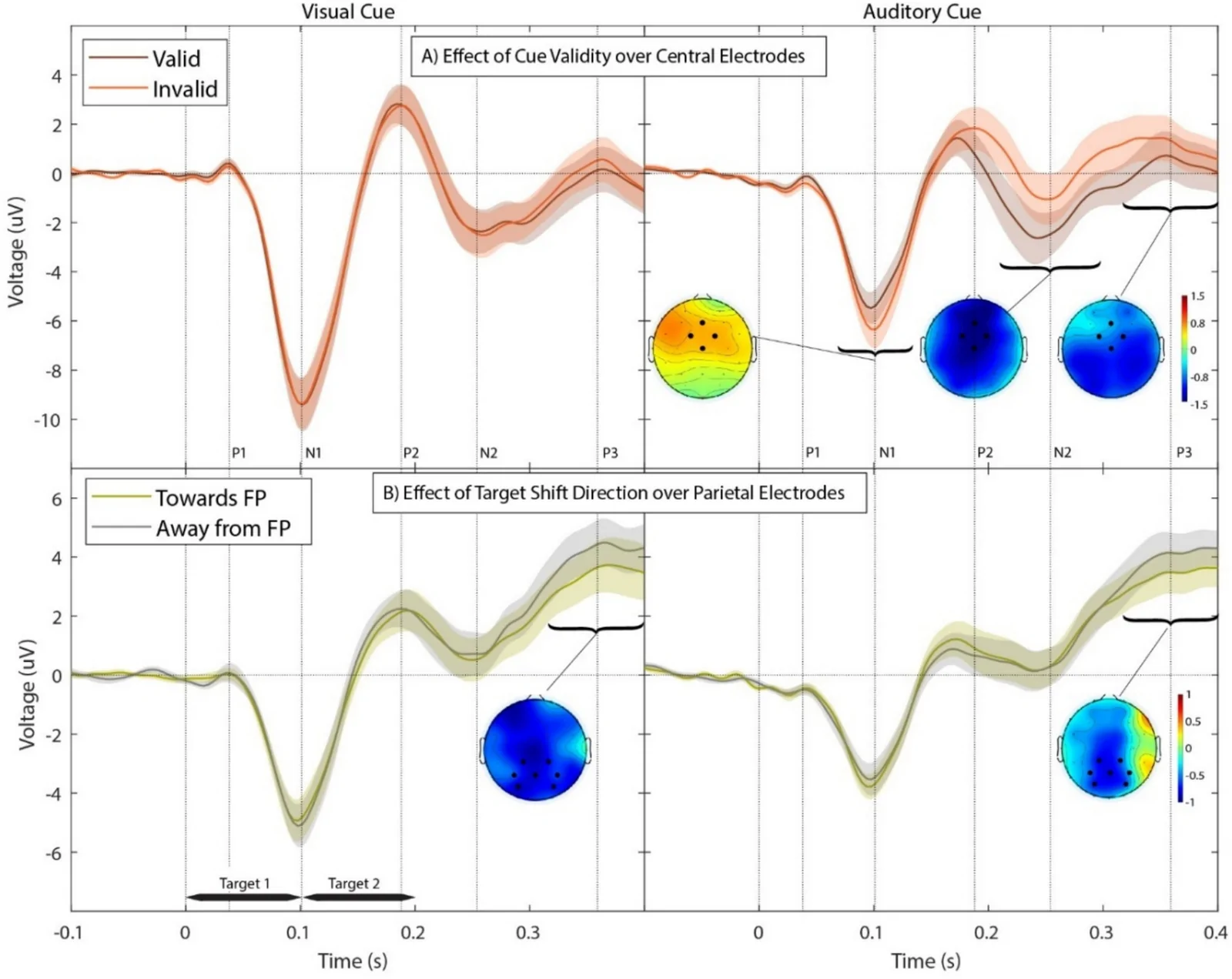
Target-elicited ERPs as a function of time, averaged over two scalp regions (central and occipital) and referenced to the target onset (indicated along the *x*-axis in the bottom left panel). **A)** Across-subject average responses over central electrodes (see bold dots in the inset topographies) for valid versus invalid cues (brown vs. orange) and visual versus auditory cues (left vs. right column). Shaded areas represent ±1 across-subject SEM. Vertical dotted lines indicate the ERP components P1, N1, P2, N2, and P3. Insets show the topography of valid–invalid differences for the component indicated by the connecting line—for N1 and N2 corresponding to significant differences across the central electrodes (for P3 the topography is shown because of a significant difference across the parietal electrodes; see text for details). **B)** Responses over parietal electrodes plotted separately for the towards versus away (beige vs. grey) from FP shift directions, using the same layout as in panel A. Insets show the topography of the significant differences of towards FP–away from FP shift direction for the P3 component. (Color figure online)

Target-elicited auditory ERPs were analyzed to identify physiological correlates of the behaviorally observed effects of cuing and eye-gaze direction on spatial discrimination. The analysis focused in two scalp topographical areas, central and parieto-occipital, previously shown to play a role in attentional cuing (Feng et al., 2014; McDonald et al., 2013). Figure 3A shows the effect of cue validity on the ERP amplitudes across the central electrodes (highlighted in the topography insets), separately for the visual and auditory cues (left vs. right panel) and temporally aligned with the target onset (indicated along the *x*-axis in the bottom left panel).

A separate exploratory repeated-measures ANOVA was performed on the across-channel average amplitude for each of the five components (P1, N1, P2, N2, P3; indicated by vertical lines in Fig. 3), with the factors of cue modality (auditory, visual), cue validity (valid, invalid), target location (central, peripheral), fixation location (left, right), and target shift direction re. FP (towards, away). Bonferroni correction was used to compensate for the multiple comparisons (considering five components and two areas, criterion p was adjusted to 0.005). Only the ANOVAs on N1 and N2 components found significant effects. The ANOVA on N1 data revealed a significant main effect of cue modality, *F*(1,13) = 43.00, *p* < 0.001, and a significant Cue Modality × Validity interaction, *F*(1,13) = 13.71, *p* = 0.003. Figure 3A shows that the target-elicited N1 for the visual cue was around −9 µV, independent of cue validity (brown and orange lines are on top of each other), while for the auditory cue it was smaller, reaching only approximately −6 µV for the invalid cue (orange) and −5 µV for the valid cue (brown). The N1 topography inset in Fig. 3A shows the auditory cue valid–invalid target-elicited N1 difference topography, with largest contrasts observed for the central and frontal electrodes, with a slight dominance on the left-hand side.

For the N2 component, only the four-way interaction of cue modality, cue validity, fixation location, and shift direction was significant, *F*(1,13) = 13.81, *p* = 0.003. A separate partial ANOVA performed on the visual-cue data found no significant effects, while a similar ANOVA on the auditory-cue data found a significant Fixation × Validity × Shift Direction interaction, *F*(1,13) = 19.86, *p* = 0.0006. This complex three-way interaction was dominated by the effect of validity (difference between valid and invalid cues was around 1.5 µV; N2 orange vs. brown graph in the right panel of Fig. 3A). Topographically, this effect of validity was concentrated around the fronto-central electrodes (N2 difference topography inset in Fig. 3A). Fixation and shift direction only had minor effects (smaller than 1 µV; data not shown) and thus were not considered further.

Figure 3B shows the effect of the target shift direction re. FP on the ERPs across the parietal electrodes, using a layout similar to Fig. 3A. As with the central electrodes, five separate ANOVAs were performed on different components of the across-channel average signal, out of which only the ANOVA on P3 found significant effects. First, the main effect of shift direction was significant, *F*(1,13) = 15.37, *p* = 0.002, with the away-from-FP signal more positive for both visual and auditory cues (see the P3 beige vs. gray graphs in Fig. 3B). The topographies of the contrast show maximums over the parietal electrodes (insets in Fig. 3B). Second, the Cue Modality × Validity interaction was significant, *F*(1,13) = 13.71, *p* = 0.003, exhibiting a pattern similar to the central-region N2 pattern shown in Fig. 3A. Specifically, the visual cue P3 values were approx. 3.6 µV independent of cue validity, while the auditory valid cue responses were slightly lower (3.1 µV) and the auditory invalid responses were considerably higher (4.4 µV) (while the ERP values are not shown for these contrasts in Fig. 3B, the P3 topography in Fig. 3A shows the valid-invalid contrast was broadly concentrated around the parietal electrodes).

#### Discussion

The effects of cue modality and validity on the central-electrode N1 and N2 (Fig. 3A) are both generally consistent with the behavioral observation that cue validity had no influence on *d′* for the visual cue but a large influence for the auditory cue. Behaviorally, a valid auditory cue caused a small improvement in spatial discrimination (compared to the average visual cue performance), while an invalid auditory cue acted as a strong distractor (Fig. 2A). The pattern for N1 only partially matches the behavior, as the visual cue response is more negative than the auditory invalid cue response, which in turn is more negative than the auditory valid response (even though a valid auditory cue would be expected to produce, if anything, a larger N1; Kennett et al., 2001; McDonald et al., 2000, 2001). In addition, the N1 response is generated in early auditory cortices (Hari et al., 1980; Virtanen et al., 1998). Thus, it appears more likely that this effect is due to neuronal adaptation resulting from repetitive stimulation. Specifically, assuming that the cortical response is suppressed and therefore weaker for a repeated presentation of a stimulus (Virtanen et al., 1998), the weaker response following the auditory versus visual cue is expected because the auditory cue sound preceded and was similar to the auditory target sound (Ahveninen et al., 2006; Butler, 1972; Jääskeläinen et al., 2004, 2007). A new result observed here is that this suppression is stronger when the auditory cue comes from the same location as the target (valid condition) than when the cue location is different (invalid condition), which is consistent with stimulus-specific adaptation (Briley & Krumbholz, 2013).

On the other hand, the N2 pattern matches behavior much better, as the auditory valid N2 is only slightly lower than the visual N2, while the auditory invalid N2 is much higher than both the visual N2 and the auditory valid N2 components (Fig. 3A). Additionally, the occipital P3 pattern matches the central N2, and even the fronto-central P3 had a tendency to be more negative for the valid than invalid auditory cue (Fig. 3A). Thus, the late components of N2 and P3 over the central and occipital scalp regions are the most likely ERP correlates of the behavioral effects of covert bottom-up attentional cuing on auditory spatial sensitivity.

The analysis of the parieto-occipital topographical area showed that the P3 component’s amplitude depends on the target shift direction re. FP, qualitatively consistent with the behaviorally observed bias away from the FP for both visual and auditory cues (Fig. 2B). However, the behavioral bias was very strong for an invalid auditory cue and negligible for a valid auditory cue, while no such effect was observed for the P3 component. Thus, it appears that this ERP component corresponds to a constant bias away from the FP. While behaviorally this effect was modulated by the cue location-dependent interference when the cue was auditory, no corresponding physiological effect of the auditory cue was observed. Auditory and visual stimuli often produce similar P3 responses in discrimination tasks (Comerchero & Polich, 1999). So, this result might be specifically related to the gaze-dependent spatial auditory discrimination bias for which, to our knowledge, there are no previous studies available.

Finally, for the P3 component, the main effect of target shift direction is additive with the Cue Modality × Validity interaction (i.e., there was no Target Shift × Cue Modality × Cue Validity interaction), suggesting that the mechanisms causing the attentional effects on this component do not directly interact with the mechanisms causing the shifts re. FP. All of these results are consistent with the P3 being a correlate of executive control (Donchin & Coles, 1988; Polich, 2007; Walhovd & Fjell, 2002), here specifically directing spatial auditory attention (along with N2) and controlling the decision about perceptual events. Importantly, to our knowledge, this study is the first one to show this component as a correlate of a fixation-dependent bias in auditory spatial discrimination, possibly related to other auditory spatial asymmetries, like the looming bias in distance perception (Baumgartner et al., 2017) or perceptual distortions in auditory space processing (Bosen et al., 2018; Brimijoin, 2018).

### Auditory cue–elicited ERPs

#### Results

To further examine the physiological mechanisms underlying the effects of cuing on auditory spatial discrimination, the final analysis focused on the responses elicited by the auditory cues (visual cues were not considered since they did not affect discrimination) before the targets were presented. The analysis examined the orienting response to the cue, possibly related to the ACOP response previously shown to correlate with the subsequent visual and auditory discrimination accuracy (Feng et al., 2014; McDonald et al., 2013). To examine whether the strength of the ACOP-related response also can act as a performance predictor in the current study, the cue-elicited ACOP-related responses were analyzed separately for the trials in which the subsequent target discrimination was correct versus those where it was incorrect (while also considering other parameters like cue validity, location, etc.). Also, the analysis considered the effect of gaze fixation on the responses to examine whether the response is encoded in a head-centered or eye-centered RF (Groh & Sparks, 1992; Lokša & Kopčo, 2023).

Previous ACOP studies used a simple setup in which the head and eyes fixated directly in front of the listener and the stimuli were only located on the left or right relative to fixation/head orientation (Feng et al., 2014; McDonald et al., 2013). Thus, the visual (eye-centered) and auditory (head-centered) representations of the space were aligned, and it was not possible to determine whether the ACOP is produced in a brain region using the eye-centered or head-centered RF. In the current study the stimuli could be on the left (−25°), central (0°), or right (+25°) locations while the eyes fixated +12.5° or −12.5°, all with respect to the head orientation (i.e., in head-centered RF). However, when expressed with respect to the eye-gaze direction (i.e., in the eye-centered RF), the current cues and targets could only be located on the left or on the right (at +12.5° or −12.5°), similar to the previous studies. Thus, the current study could examine whether the ACOP-related response, if observed, would be more aligned with the eye-fixation-independent head-centered RF (consistent with the auditory space representation of the auditory stimuli used here) or the eye-centered representation (consistent with the visual representation in the occipital brain areas where this response is assumed to originate).

**Fig. 4:**
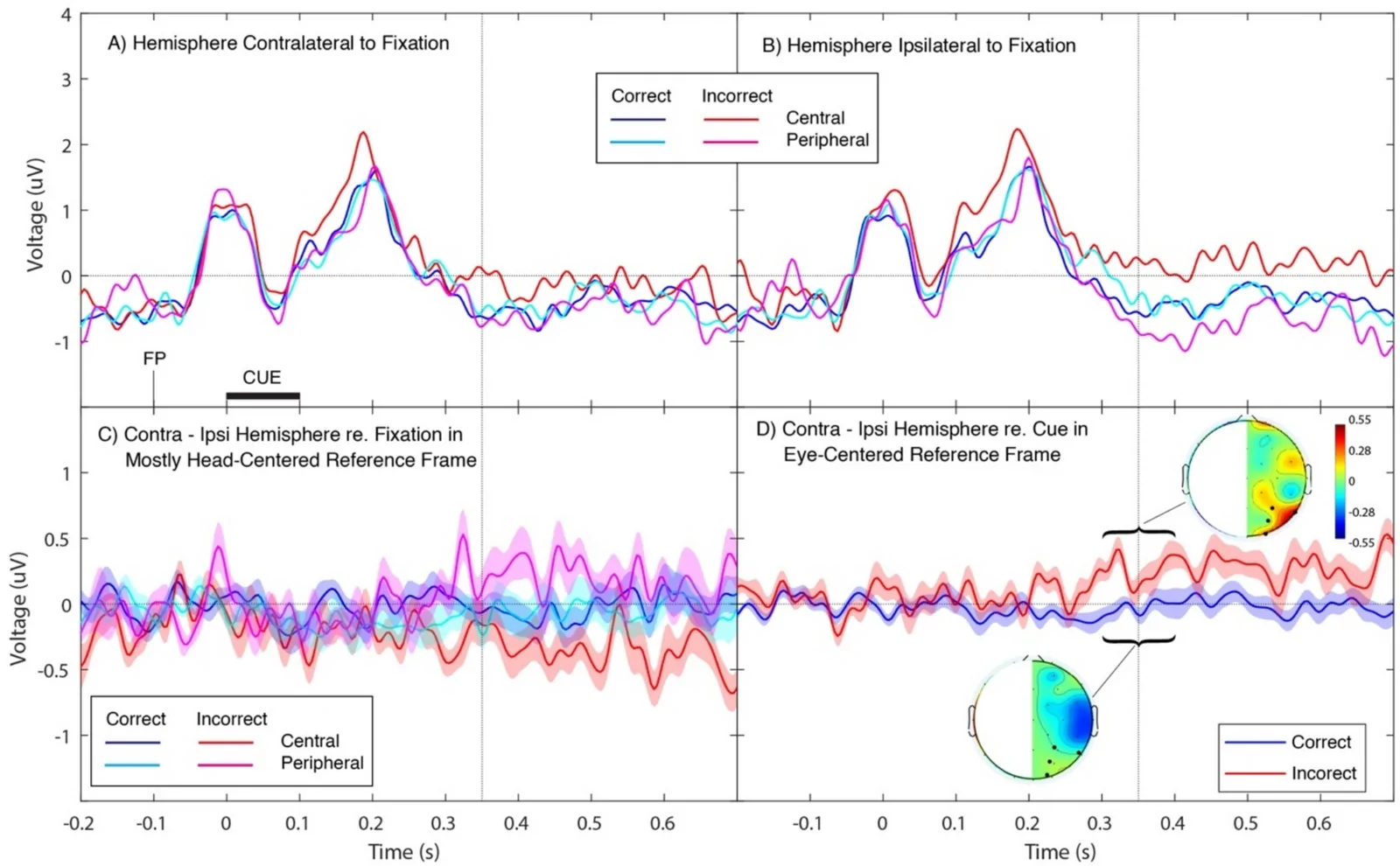
Auditory cue-elicited ERPs over occipital electrodes shown separately for each hemisphere (**A, B**) and as a hemispheric difference (**C, D**). The responses are always averaged across 4 occipital electrodes (the right-hemisphere electrodes are highlighted in the insets, while the corresponding electrodes considered in the left hemisphere are not shown). The traces in the upper panels show the across-subject mean ERP for all combinations of correctness (correct vs. incorrect) and cue position (central vs. peripheral), plotted separately for contralateral (**A**) or ipsilateral (**B**) hemisphere re. fixation on data averaged across fixation location and validity. **C)** The hemispheric difference (contra – ipsi hemisphere re. fixation) responses obtained by subtracting the respective data from panel B from those from panel A, representing the ACOP response in a mixed head-centered and eye-centered reference frame. **D)** Hemispheric difference (contra – ipsi hemisphere re. cue location in eye-centered reference frame) obtained after encoding the conditions with respect to the eye fixation and referencing the hemisphere to the cue location (e.g., the red trace in panel D was obtained as the average of the purple and sign-flipped red traces from panel C). (Color figure online)

The ACOP is a hemispheric differential response, therefore a group of left–right symmetrically placed occipital electrodes was considered for each hemisphere (highlighted for the right hemisphere in the insets of Fig. 4) and the analysis focused on the time period 300–400 ms post cue onset (indicated in all panels of Fig. 4 by a vertical line at 350 ms), similar to the previous studies. Fig. 4A–B show the average within-hemisphere responses, respectively, for the hemisphere contralateral and ipsilateral to fixation. Each line represents the response for one combination of correctness and cue location on data averaged across cue validity and FP location, as these two factors did not produce any significant main effect or interaction (see the analysis below) and time-aligned with respect to the cue onset (indicated along the *x*-axis). While these graphs show strong early (*t* < 300 ms) positive and negative responses to the cue and even to the FP (which preceded the cue), these deviations are relatively similar across the hemispheres and across different conditions. However, starting at approximately 300 ms, the within-hemisphere responses return to zero, while the separation between different conditions and between the hemispheres becomes larger.

To directly evaluate the ACOP-related response, a repeated-measures ANOVA was performed on the contra − ipsi hemisphere responses (i.e., panel B minus panel A, shown in panel C) over the 300–400-ms period with the factors of discrimination correctness (correct/incorrect), validity (valid/invalid), cue position (peripheral/central), and fixation (left/right). It found a significant main effect of cue position, *F*(1,13) = 9.86, *p* = 0.008, and a significant interaction of correctness and cue position, *F*(1,13) = 8.47, *p* = 0.012. Figure 4C shows the differential plot paralleling this ANOVA, with each line corresponding to the difference between the respective lines from panels A and B. It shows that while for the correct trials there is very little differential effect (blue and cyan lines are near zero), for the incorrect trials starting at around 300 ms, the difference became positive for the peripheral cues (magenta line) and negative for the central cues (red). This confirms that the ACOP-like response correlates with the subsequent discrimination accuracy, but it is not always a positivity when hemispheres are encoded relative to fixation and it only is visible for the incorrect responses.

The RF used to encode the data in Figure 4C is a mixture of head-centered and eye-centered, as it considers the head-centered location of the FPs and partially also of the cues (which are simultaneously also referenced to the fixation) while the hemispheres are referenced to the fixation. To evaluate the data in a purely eye-centered RF, the conditions were recoded such that, for each cue, only its location relative to the fixation was considered (i.e., left vs. right) and the hemisphere was expressed as ipsi/contralateral with respect to that cue’s eye-centered location (e.g., for a trial in which the fixation was at +12.5° and the cue was at 0°, the cue was encoded as being on the left, and the left hemisphere was considered to be ipsilateral while the right hemisphere was contralateral). A repeated-measures ANOVA was performed on the hemispheric difference data with this encoding, using the factors of discrimination correctness (correct/incorrect), validity (valid/invalid), cue position (left/right), and fixation (left/right). This ANOVA found only a significant main effect of discrimination correctness, *F*(1,13) = 8.47, *p* = 0.012, paralleling the Correctness × Cue Position interaction of the preceding ANOVA. Figure 4D plots the corresponding data for the correct and incorrect trials (these curves are derived from those in panel C; e.g., the red line in Fig. 4D is obtained as an average of the magenta line and the sign-inverted red line from Fig. 4C). Notably, these data are much less noisy and their error range (shown by light red and blue bands corresponding to ±1 SEM) is much smaller than those in panel C. While no effect is observed initially, in the 300–400 interval the incorrect trial values were around 0.35 µV (red), while the correct trial values stayed around 0 µV (blue). Interestingly, this difference between the red and blue lines appears to be approximately constant also for the reminder of the responses, up to 700 ms. The inset topography connected to the red line in Fig. 4D shows the hemispheric contrasts for the incorrect trials, clearly demonstrating that the ACOP response is concentrated around the four occipital electrodes, while the inset connected to the blue line shows no activation in that region for the correct trials. Overall, this analysis shows that the ACOP is encoded in eye-centered RF, but only for the incorrect trials, and that it is not dependent on the cue validity or its location in the head-centered RF.

Finally, a similar analysis was conducted for visual cues to determine whether the ACOP-like response in the eye-centered reference frame is specific to auditory cues or generalizes across modalities. The same occipital electrodes and 300–400 ms time window were used, and the contra-minus-ipsi difference was computed (see Supplementary Fig. 1). A repeated-measures ANOVA (Correctness × Validity × Cue Position × Fixation) with the data were recoded into the eye-centered reference frame yielded no significant main effects or interactions (all *p* values ≥ 0.07). Thus, unlike the auditory-cue analysis, which showed a clear eye-centered ACOP-like response differentiating incorrect from correct trials, the visual-cue responses did not exhibit a statistically reliable eye-centered effect under the current experimental conditions.

#### Discussion

This analysis showed that, consistent with previous studies (Feng et al., 2014; McDonald et al., 2013), an ACOP-like response was evoked by the auditory cue in the current experiment in which auditory stimuli were used as targets. However, the response was only observed for the incorrect trials, independent of cue validity, while the previous study (Feng et al., 2014) mostly observed it for valid correct trials. This inconsistency is most likely due to differences in the experimental design between the studies, the most prominent of which is the target modality (Feng et al., 2014, used a visual target). Also, the previous study reported ACOP only in the time period of 300–400 ms, while the current study shows the response continues for as long as 700 ms, possibly due to a longer time window used after the cue in the current study.

Finally, the current study shows that the ACOP-like response observed here is most likely encoded in the eye-centered RF, consistent with the assumption that it is generated in the visual occipital brain regions. This finding of an auditory-cue-elicited response in visual cortex aligns with a large body of work demonstrating robust crossmodal links to early visual cortex (Driver & Noesselt, 2008), showing that auditory spatial information can modulate visual sensory processing even in the absence of visual stimulation. Importantly, the auditory spatial information about the cue must be recoded from the natively head-centered representation to the eye-centered representation observed in the ACOP when the two RFs are misaligned as in the current study. The finding that this signal is encoded in eye-centered coordinates highlights a potential computational burden. For it to influence auditory processing, the signal must be transformed from its native eye-centered representation back to head-centered coordinates, a process known to involve areas such as the parietal cortex (Cohen & Andersen, 2002; Pouget et al., 2002). This recoding demand is likely a source of the interference we observe on invalid trials, the system may engage in costly coordinate transformations that compete with or disrupt target processing.

This recoding might be the main driver of the differences observed in this and the previous studies, which always kept the eye-centered and head-centered RFs aligned and static by employing left–right symmetrical setups and eyes fixated in the center. Additional studies would be needed to explore whether the driving mechanism of the current results is related to factors like compensatory responses or recoding costs.

To determine whether the opposite-polarity ACOP responses observed for peripheral and central cues on incorrect trials were statistically reliable, we computed the mean contra-minus-ipsi amplitude over the 300–400-ms time window for each condition and tested these values in one-sample *t* tests against zero. For peripheral cues, the mean difference was significantly positive (*M* = 0.231 µV, *SD* = 0.389), *t*(13) = 2.22, *p* = 0.045. For central cues, the mean difference was significantly negative (*M* = –0.281 µV, *SD* = 0.404), *t*(13) = –2.60, *p* = 0.022. These results confirm that, on incorrect trials, peripheral cues elicited a reliable contralateral positivity, whereas central cues elicited a reliable contralateral negativity when hemispheric difference was computed relative to fixation. This opposing pattern is consistent with an eye-centered reference frame for the ACOP, as central cues (0° relative to head) are lateralized relative to gaze and thus produce a contralateral response that appears inverted when incorrectly referenced to head-centered fixation.

Finally, while a response like the previously reported ACOP is observed here, it is not clear whether it has any functional relevance for the auditory cuing effects on auditory targets in the current study, as the ACOP signal would need to be recoded back to the head-centered representation in order to correctly affect auditory processing of the target.

## General discussion

A combined behavioral and EEG experiment investigated the effects and neural mechanisms of involuntary spatial orienting on auditory spatial discrimination. The behavioral experiment found that, for intramodally controlled attention, an invalid auditory cue reduced sensitivity and induced a strong bias in responses, while a valid auditory cue did not affect performance significantly. For crossmodal attentional control, there was no visual valid-versus-invalid cuing effect on sensitivity, while the responses were always biased away from the FP. In ERP analysis, the target-elicited central-electrode N2/parietal-electrode P3 responses matched the behavioral effect of cue validity and modality on discrimination. Finally, a late auditory cue-elicited hemispheric difference response was observed over the occipital electrodes for the incorrect trials, independent of cue validity. This ACOP-like response predicts the accuracy of subsequent responses and appears to be encoded in the eye-centered RF.

The current behavioral results confirmed the hypothesis that automatic orienting influences auditory spatial discrimination sensitivity intramodally, but not crossmodally, when the eyes are fixated at a neutral location throughout the trial. This result is consistent with previous studies in which visual sensitivity was shown to be more affected intramodally than crossmodally (Blurton et al., 2015; Buchtel & Butter, 1988; Spence & Driver, 1994). However, the effect in the current study appears to reflect distraction by invalid cues, not an enhancement of sensitivity for valid cues. And it was accompanied by strong biases for invalid auditory as well as visual cues. More studies are needed to examine the interaction between eye gaze shifts (Maddox et al., 2014) and the automatic orienting effects (current study).

Maddox et al. (2014) proposed a model in which eye gaze shifts are accompanied by shifts in the receptive fields of binaural auditory spatial neurons that could explain the changes in discriminability observed in their study. Similar changes in the receptive fields of binaural neurons might be induced by automatic orienting. However, it is likely that other mechanisms are also involved (e.g., the “inhibition of return” is also a detrimental attentional phenomenon that operates on the timescales of 500–3,000 ms, comparable with the current study; Posner & Cohen, 1984). Future studies could provide more information about the combined effects of eye gaze and automatic orienting by examining other combinations of cue/target locations (e.g., left–right symmetrical conditions) and cue/target stimulus types (e.g., stimuli that are less similar than in the current study).

Several ERP components of the EEG responses to the target correlated with the behavioral effects. This suggests that the neural mechanisms of repetition adaptation (central electrode N1), automatic interference (central N2/parietal P3), and executive control (parietal P3) likely affected observed performance. Most previous EEG studies of auditory spatial discrimination used the oddball paradigm. They observed responses mainly in the form of mismatch negativity (MMN; Altman et al., 2005; Deouell et al., 2006; Lewald et al., 2018; Matthews et al., 2007) and in the P3 component (Getzmann et al., 2022; Polich, 2007; Verleger et al., 2005), likely driven by the “novelty” aspect of the oddball stimulus. The responses in the current study should not be contaminated by such factors, only reflecting activation due to auditory spatial discrimination and attentional processing. Furthermore, the current study used a full simulation of HRTFs, likely enhancing attention-related ERP responses (Deng et al., 2019). Finally, the EEG response to the target confirms that the covert bottom-up orienting of attention to sounds relies on neural biasing mechanisms in early visual processing (Cate et al., 2009; Störmer et al., 2019) and suggests that the auditory cue stimuli are converted into the visual eye-centered representation for this orienting. However, the cue-related response observed here was more extended in time than the typical ACOP response, perhaps reflecting the additional processing involved in conversion of the RFs.

This study has some limitations. First, the findings should be considered in light of the sample size. With only thirteen participants, the statistical power to detect smaller effects is limited. This is particularly relevant for the interpretation of null results, such as the absence of significant higher-order interactions in our ANOVA analyses. It is possible that effects of smaller magnitudes exist but were not detectable in the current sample. Second, the fixed order in which the blocks were presented within a session, might have introduced some systematic biases despite the counterbalancing across sessions. One methodological consideration is that the fixation point appeared only 100 ms before the cue. Although this brief interval could in principle influence attentional allocation, it is highly unlikely as the FP was entirely predictable in both its timing and location and participants in general maintained fixation at this location throughout each block, making the FP a stable and easily ignorable event. Moreover, if the FP appearance had any effect, it would be the same for valid and invalid and auditory and visual cues.

In summary, this study showed that involuntary orienting affects auditory spatial discrimination when gaze is fixed at a neutral location, but that there still is an effect of gaze direction on performance. Several ERP responses to the cue and the target can be linked to behavioral performance. Additional studies are needed to further examine the behavioral and neural mechanisms underlying intramodal and crossmodal covert bottom-up auditory orienting in auditory spatial discrimination.

## Supplementary Information

Below is the link to the electronic supplementary material.

Supplementary file1 (DOCX 233 KB)

## Data Availability

The data supporting the findings of this study are openly available on Zenodo [10.5281/zenodo.17035882]. The repository includes both trial-level behavioral data and raw EEG data associated with the present studies. The studies reported in this manuscript were not preregistered.

## Code availability

The experiment and analysis code will be made available upon reasonable request. The experiment was not preregistered.
