# Supplementary material for: Modality-dependent bottom-up attention and eye gaze direction affect auditory spatial discrimination
Source: Atten Percept Psychophys. 2026 Jul 9;88(6):158. doi: 10.3758/s13414-026-03305-9 (PMC13350116; doi:10.3758/s13414-026-03305-9)
Supplement: Supplementary file 1 — Supplementary file1 (DOCX 233 KB) [file 13414_2026_3305_MOESM1_ESM.docx]

**Supplementary Materials**


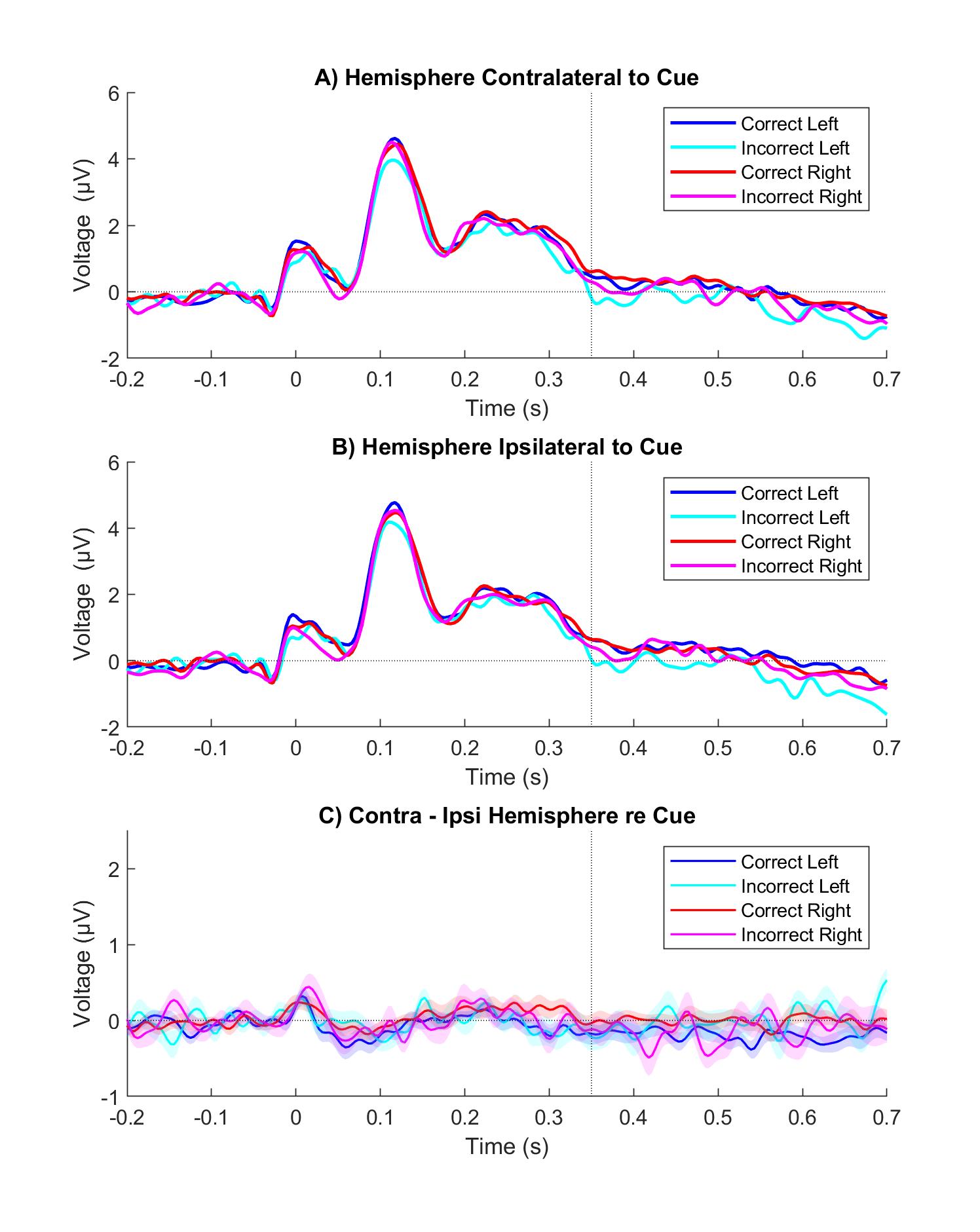


**Supplementary Figure 1.**

Visual cue–elicited ERPs over occipital electrodes, shown separately for each hemisphere (A, B) and as a hemispheric difference (C). The responses are always averaged across 4 occipital electrodes (shown in Fig. 4). The traces in the upper two panels show the across-subject mean ERP for all combinations of correctness (correct vs incorrect) and cue position (left vs right), plotted separately for contralateral (A) or ipsilateral (B) hemisphere re. cue location in eye-centered reference frame on data averaged across fixation location and validity. location and validity. C) The hemispheric difference (contra – ipsi hemisphere re. cue) responses obtained by subtracting the respective data from panel B from those from panel A.
